# Highly Sensitive Detection of Melamine Using a One-Step Sample Treatment Combined with a Portable Ag Nanostructure Array SERS Sensor

**DOI:** 10.1371/journal.pone.0154402

**Published:** 2016-04-27

**Authors:** Jie Cheng, Xiao-Ou Su, Yue Yao, Caiqin Han, Shi Wang, Yiping Zhao

**Affiliations:** 1 Institute of Quality Standards and Testing Technologies for Agro-products, Chinese Academy of Agricultural Sciences, Beijing, China; 2 School of Physics and Electronic Engineering, Jiangsu Normal University, Jiangsu, China; 3 Department of Physics and Astronomy, University of Georgia, Athens, United States of America; University of Gdansk, POLAND

## Abstract

There is an urgent need for rapid and reliable methods able to detect melamine in animal feed. In this study, a quick, simple, and sensitive method for the determination of melamine content in animal feed was developed using surface-enhanced Raman spectroscopy on fabricated Ag nanorod (AgNR) array substrates with a one-step sample extraction procedure. The AgNR array substrates washed by HNO_3_ solvent (10^−7^ M) and methanol and showed the good stability within 6 months. The Raman shift at △*ν* = 682 cm^−1^ was used as the characteristic melamine peak in the calculations. Sufficient linearity was obtained in the 2–200 μg·g^−1^ range (*R*^2^ = 0.926). The limits of detection and quantification were 0.9 and 2 μg·g^−1^, respectively. The recovery rates were 89.7–93.3%, with coefficients of variation below 2.02%. The method showed good accuracy compared with the tradition GC-MS analysis. This new protocol only need 2 min to fininsh the detection which could be developed for rapid onsite screening of melamine contamination in quality control and market surveillance applications.

## Introduction

Melamine (1,3,5-triazine-2,4,6-triamine, C_3_H_6_N_6_) is a nitrogen-rich compound commonly used to produce melamine-formaldehyde resins, which are utilized as coatings, glues, laminates, and heat-tolerant polymers [1–4]. It is implicated in the pet and human food recalls in 2007 [5] and in the global food safety scares in 2008 involving milk and milk-derived products [6–8]. In those food safety incidents, melamine was deliberately added to animal feed to elevate the measured protein content. Although various toxicology studies have found that melamine toxicity in mammals is very low [9], a recent study revealed that when melamine and cyanuric acid co-exist, melamine cyanurate crystals form, which show higher toxicity [5].

Several analytical methods for the detection of melamine in feed have been established, such as liquid chromatography-tandem mass spectrometry (LC-MS/MS), which is the analytical method currently used by the Food and Drug Administration (FDA) [10], and gas chromatography/mass spectrometry (GC/MS), which is used in China to quantify melamine in feed [11]. These methods require time-consuming clean-up steps and high-cost instruments. However, some screening techniques have been developed for melamine detection, such as enzyme-linked immunosorbent assay (ELISA) [12–14] and near-infrared reflectance spectroscopy (NIR) [15]. The ELISA technique shows limitations due to a high rate of "false positive" results, and NIR is limited by relatively poor sensitivity. Therefore, it is of critical importance to develop simpler, accurate, and sensitive fast screening methods for melamine detection in feed.

Surface-enhanced Raman spectroscopy (SERS) is an attractive technique that applies chemical and electromagnetic mechanisms to enhance the Raman scattering signals of target molecules adsorbed on noble metallic nanostructures [16,17]. Signals are normally enhanced 10^4^~10^6^ times, sometimes even reaching 10^14^ times [18]. The trace analysis of a single molecule in real samples using SERS often has two key features. Firstly, the sample clean-up process is simple and affords high recovery. Secondly, the ideal SERS substrate, which is both uniform and stable, *e*.*g*., metal nanostructure arrays, leads to high reproducibility [19]. In previous studies, our group prepared and applied colloidal Au in the SERS detection of melamine, including the development of convenient sample preparation by liquid-liquid extraction [20]. In the current study, we acquired the Raman and SERS spectra of melamine, and compared the experimental data with theoretical calculations using density functional theory (DFT). We also modified the sample clean-up method and fabricated SERS-active Ag nanorod (AgNR) array substrates using oblique angle deposition (OAD) to detect melamine in feed. The modified clean-up method reduced the detection time and the active substrate was much more controllable and stable.

## Experimental

### Materials

Melamine was purchased from Sigma-Aldrich Co., Ltd. (Shanghai, China). Ag (99.999%) and Ti (99.995%) pellets were obtained from Kurt J. Lesker (Clairton, PA). Methanol and all other chemicals were of analytical grade and obtained from Sinopharm Chemical Reagent Co., Ltd. (Beijing, China). Stock solutions of melamine (2 mg·mL^−1^) were stored in the dark at -18°C for 3 months prior to analysis, and their subsequent dilutions (2, 10, 50, 100, and 200 μg·mL^−1^) were prepared in deionized water (18.2 MΩ·cm) for SERS analysis. All solutions were allowed to reach room temperature 2 h prior to use.

Five types of commercial feed (formulated, complete, concentrated, mixed, and compound premix) were obtained locally (collected by the Chinese National Feed Supervision from 2012 to 2014) and ground to 40-mesh powders.

### Fabrication of AgNR substrates

SERS-active AgNR array substrates were fabricated using oblique angle deposition (OAD) in a custom-built electron beam evaporation system, as previously described [21,22]. Briefly, all glass slides were cleaned using Piranha solution (80% sulfuric acid, 20% hydrogen peroxide), rinsed with deionized water, dried with an N_2_ flow, and loaded into the deposition chamber above the source material. Under high vacuum (<10^−6^ Torr), 20nm Ti and 200nm Ag were deposited onto the glass slides at a normal incidence angle and deposition rates of 0.2 and 0.3nm·s, respectively. Then, the substrate surface normal was rotated to 86° with respect to the incident vapor direction, and Ag deposition continued at a rate of 0.3nm·s. The last OAD step yielded a film of aligned nanorods ~900nm in length and ~100nm in rod diameter, with a tilting angle of ~73°with respect to the substrate normal [21,23]. Prior to each SERS measurement, the as-deposited AgNR substrates were cleaned for 2min using a custom-built inductively coupled radio frequency (RF) plasma chamber, in order to remove the organic contaminants accumulated during fabrication and storage. The chamber was operated at 30 W under a constant flow of ultrapure Ar with a chamber pressure of ~600 mTorr [24].

The detail of the deposition configuration and conditions can been seen in S1 Text and S1 Fig.

### Sample pretreatment

The feed samples were spiked by mixing blank commercially available animal feed (formulated, complete, concentrated, mixed, and compound premix) with melamine. For example, the feed (1 g) was spiked with melamine stock solution (10 μL, 2 mg·mL^−1^), such that the melamine concentration of the feed sample was 20 μg·g^−1^. All of the spiked samples were extracted with methanol. Melamine was extracted at a ratio of 1 g sample to 4 mL methanol. The samples were sonicated for 1.5 min at 25°C and centrifuged at 13,500 rpm for 20 s. For SERS detection, 2 μL supernatant was used.

### SERS measurement

The SERS spectra were measured on a portable Raman spectrophotometer (CTRS-2200-DC, assembled by our research group) equipped with a 785 nm diode laser, a spectrometer, and an integrated fiber-optic Raman probe for excitation. The fiber-optic Raman probe was fixed above the substrate and the substrate was placed on the microscope stage which was purchased from SCI-Bridge Co., Ltd. (Beijing, China). The microscope stage was operated manually to adjust the position of the laser beam focus. In order to place the optimum positon of the Raman fiber-optic probe we scan the melamine standard solution (C = 1μg·mL^−1^) before every experiment. All the Raman test was operated in the dark room. The substrate surfaces were cleaned using HNO_3_ solvent (10^−7^ M) and methanol. The melamine solution or sample (2 μL) was applied to the substrate surface, allowed to dry under ambient conditions, and then the SERS spectra were acquired from nine randomly selected locations with a laser power of 100 mW and an exposure time of 10s.

The SERS detection flow diagram was shown in S2 Fig.

### DFT calculations

The theoretical Raman spectra of melamine were calculated using the Gaussian 09W DFT package. The DFT calculations were based on Becke’s three-parameter exchange function (B3) [25] with the dynamic correlation function of Lee, Yang, and Parr (LYP) [26]. The molecular geometries of melamine were optimized using the hybrid B3 (exchange) and LYP (correlation) function (B3LYP) in conjunction with a modest 6–311g** basis set [27].

### Data analysis

The SERS spectral data were analyzed using Origin 8.5 (OriginLab, Northampton, MA) from 350 to 1800cm^−1^. The baseline shift was offset using the Savitzky–Golay second derivative transformation [28]. Pre-processing algorithms, such as polynomial subtraction and smoothing, were employed to analyze the data. The key parameter was set as follows: Savitzky-Golay method; Points of Windows: 7; Polynomial Order: 2.

## Results and Discussion

### DFT-calculated and experimental Raman and SERS spectra of melamine

**Fig 1:**
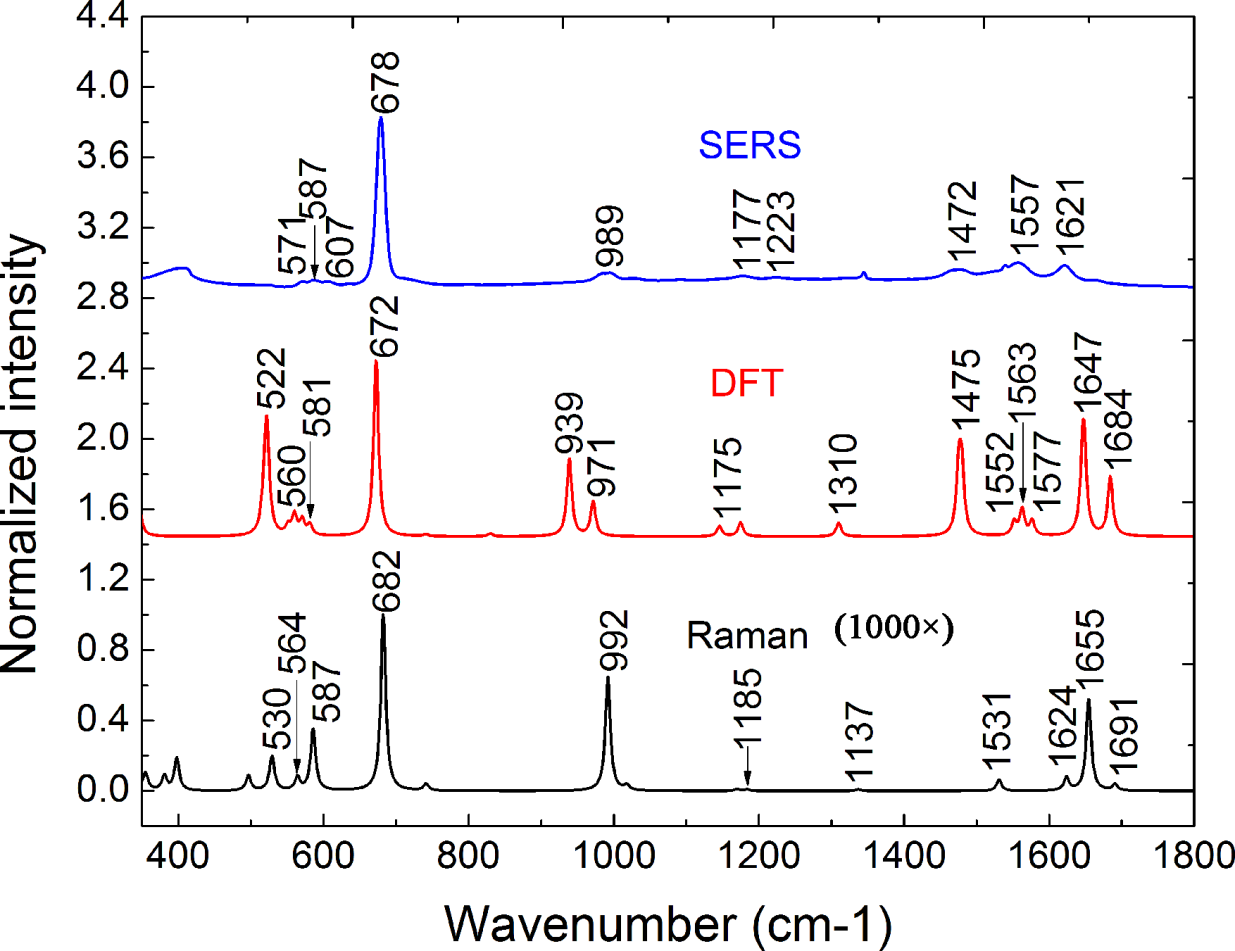
DFT-calculated and experimental Raman and SERS spectra of melamine. The Raman spectra calculated by DFT (black, 1000×) and corresponding bulk Raman (red) and SERS (blue) spectra of melamine. Spectra are normalized to the most intensive peaks and offset for clarification.

The major peaks revealed by DFT-based quantum chemical calculations (S1 Table; S2 Text) agree well with the bulk Raman spectra acquired from the melamine powder (Fig 1). However, several noticeable discrepancies between the DFT-calculated and experimental spectra are observed, including the disappearance of some DFT-calculated peaks and peak intensity changes, which can be attributed to the inherent drawbacks of DFT[27], as well as the merging of adjacent experimental Raman peaks. The Raman spectrum of melamine obtained in this study is consistent with those acquired in previous reports, with minor differences in relative peak intensities [29–31], which is likely caused by the use of different excitation powers or wavelengths. The SERS spectra of melamine (200 μg·mL^−1^) obtained on the AgNR substrates further demonstrate the effect of peak merging, as fewer peaks are observed (blue curves in Fig 1). Differences between the Raman and SERS spectra of melamine are expected since the molecules on the SERS substrate adopt different conformations from those in the crystalline powder. Peak shifts could also originate from interactions between the melamine molecules and the Ag substrate [32].

### Characteristic melamine peaks in real feed samples

**Fig 2:**
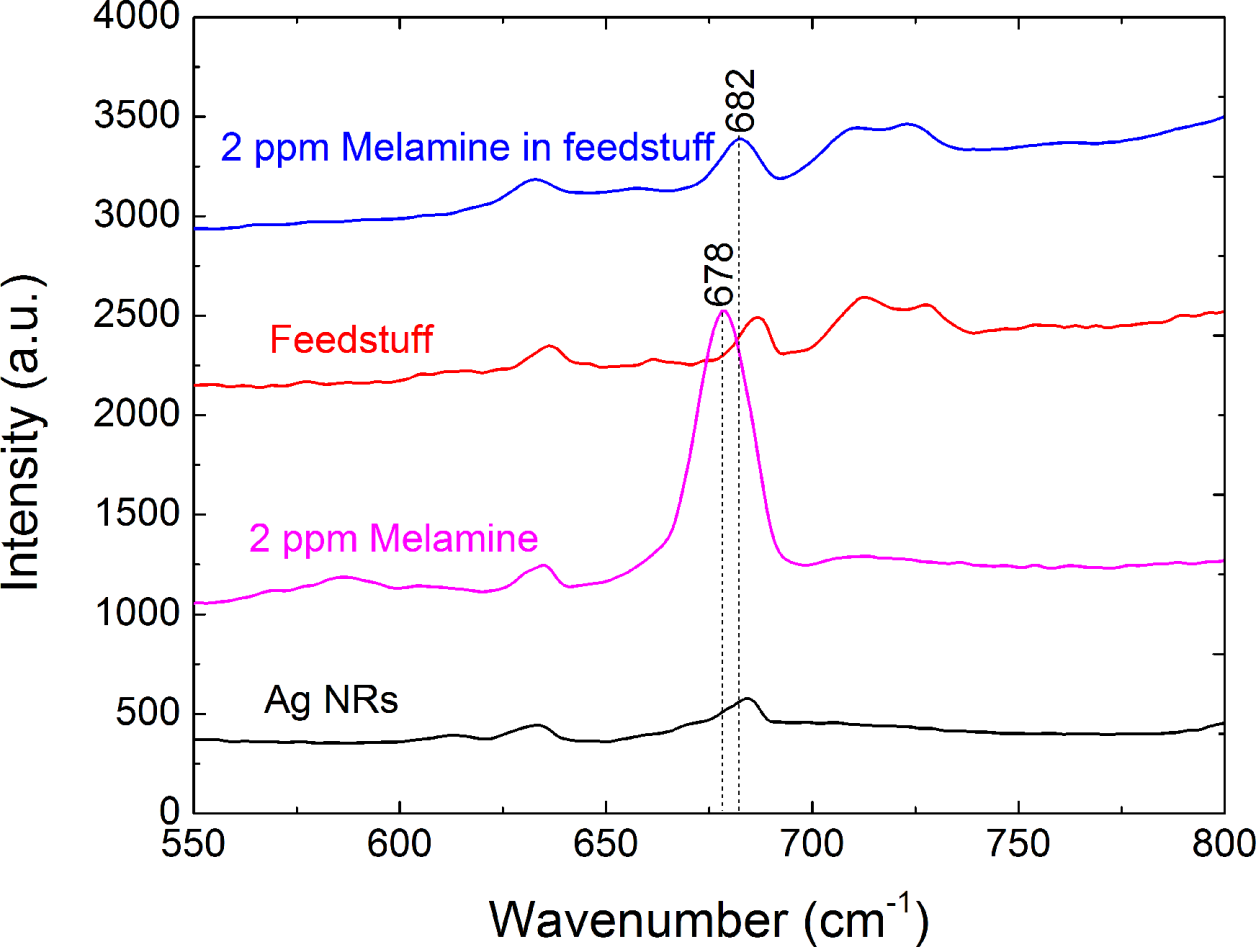
Raman spectra of samples. Raman spectra of the prepared AgNR substrates, blank feed (after sample treatment), melamine standard solution (2 μg·mL^−1^), and blank feed (after sample treatment) spiked with a standard melamine solution (spiked concentration, 2 μg·g^−1^).

**Fig 3:**
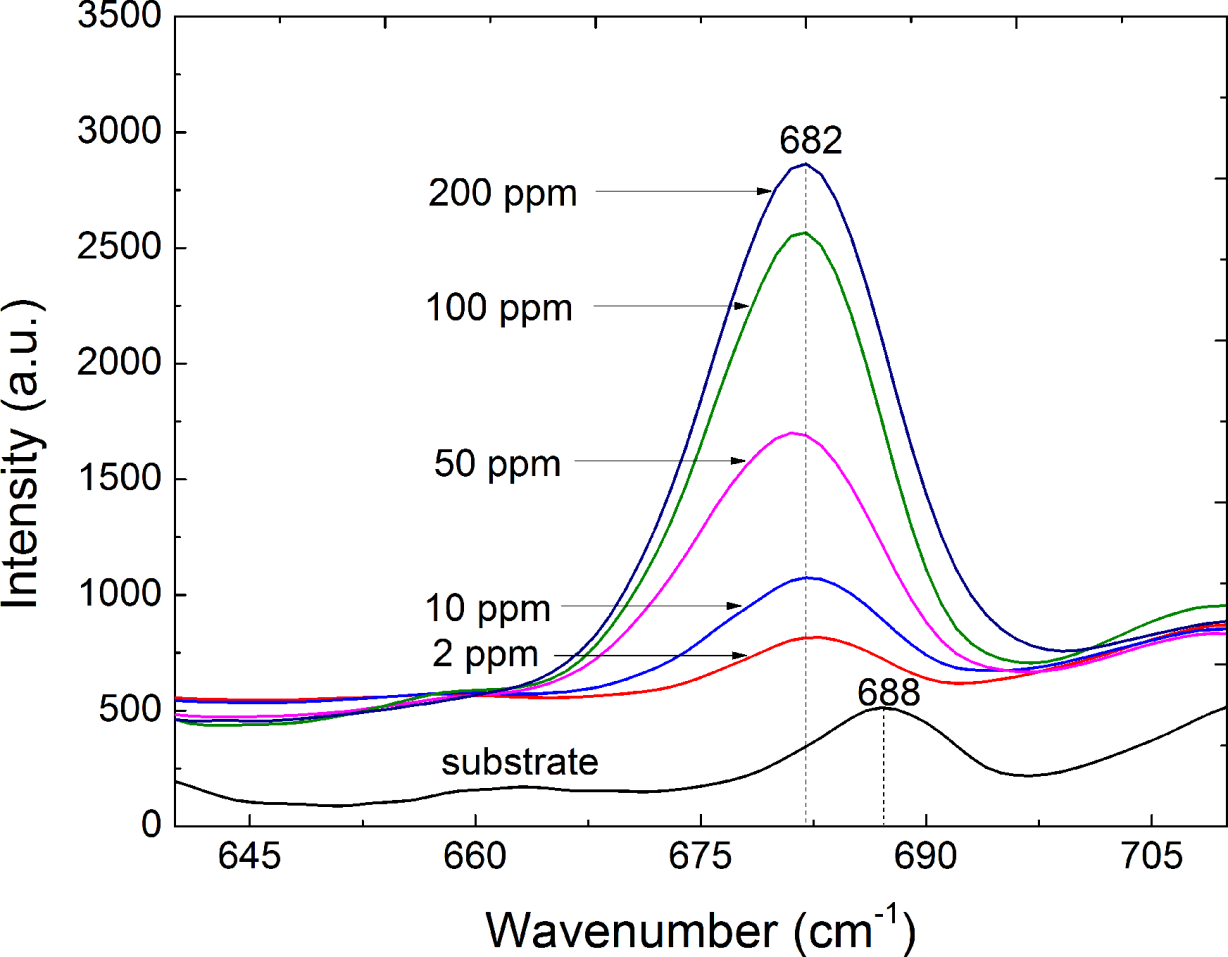
SERS spectra of spiked feed samples. SERS spectra of feed samples spiked with different concentrations of melamine (blank, 2, 10, 50, 100, and 200 μg·g^−1^).

**Fig 4:**
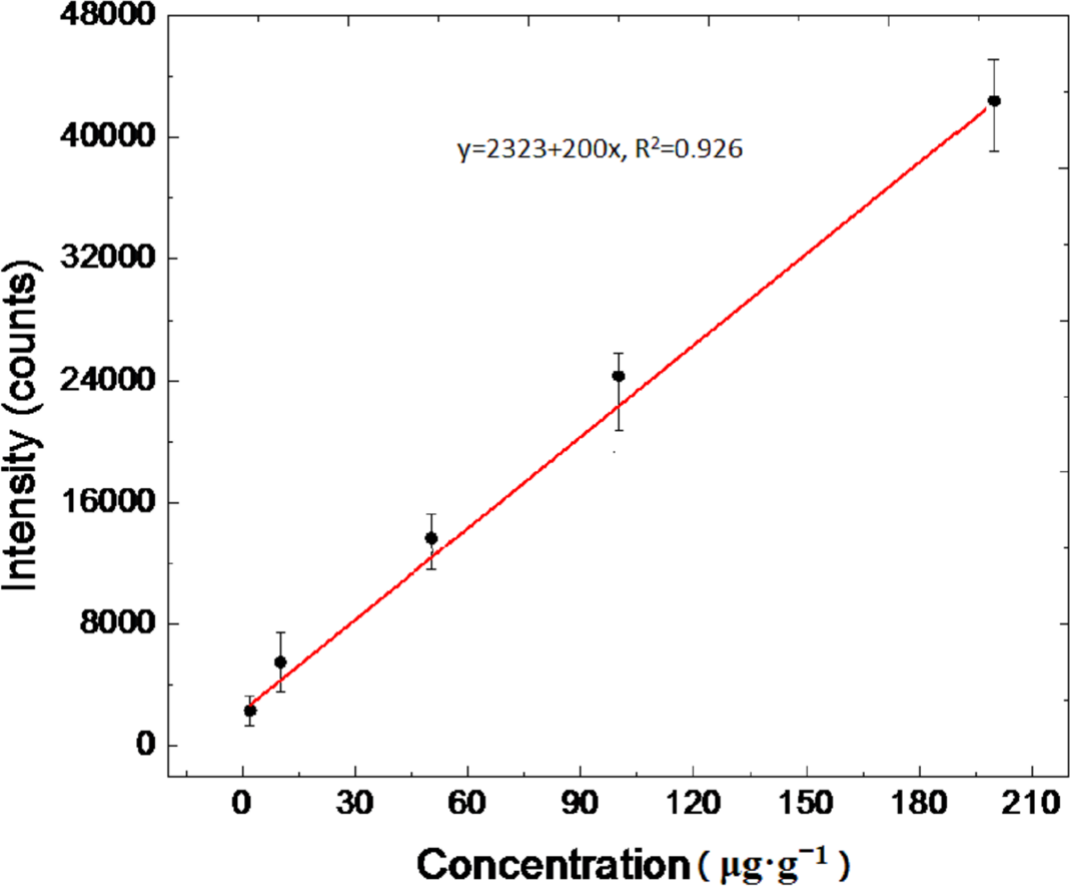
Feed-response curves for spiked feed samples. Melamine concentration fortified in feed-response curves for feed samples spiked with melamine in the range 2−200 μg·g^−1^.

To determine the characteristic peaks of melamine in real feed samples, we investigated the Raman spectra of the prepared AgNR substrates, blank feed (after sample treatment), melamine standard solution (2 μg·mL^−1^), and blank feed (after sample treatment) spiked with a standard melamine solution (2μg·g^−1^). The most intense Raman peak associated with the melamine standard solution is observed at 678 cm^−1^ (Fig 2). In the actual feed samples, the characteristic peak shifted from 678 to 682 cm^−1^ (Fig 2), which may be caused by matrix effects. These results contrasted with those of the previous study [18], where the characteristic peak of melamine was observed at △ν = 707 cm^−1^. This is likely due to the different configurations and types of active substrates used for the colloidal Au in the previous study and AgNR array substrates here. In the spectra of the prepared AgNR substrates using blank feed, the peak at 682 cm^−1^ is not observed. And compared with the previously reported method[20], we need not choose the normalized peak to realize the calculation. The peak at 682 cm^−1^ is the ideal characteristic melamine peak in the feed matrices for detection/quantification. The SERS spectra of the feed samples spiked with known concentrations of melamine (0, 2, 10, 50, 100, and 200 μg·g^−1^) (*n* = 6) are shown in Fig 3. Linear regression analysis (R^2^ = 0.926) was applied to the relationship between the Raman peak intensity at 682 cm^−1^and the different concentrations of melamine in the fortified feed (Fig 4) in the range 2–200 μg·g^−1^.

### Optimization of sample treatment

In our previous report [20], a rapid, selective, and sensitive method to determine the melamine content in animal feed was developed using SERS on aggregated 55 nm Au nanoparticles with liquid-liquid extraction sample preparation. Briefly, butyl alcohol was used as the initial extraction solvent, and liquid-liquid extraction was performed twice using HCl (pH 3–4) and 6:1 (v/v) *n*-butyl alcohol/ethyl acetate. The recovery rates were 82.5–90.2% with coefficients of variation below 4.02%. In this study, we used the above clean-up method for the prepared AgNR array substrates to detect melamine in real feed samples. No characteristic peak at △*ν* = 682 cm^−1^wasobserved. Therefore, the clean-up method described above for the AgNR array substrates was unfit for melamine detection in real feed samples. In this study, we did not use aqueous alkali or acid to adjust the pH value, based on the pK_a_ of melamine. We developed a new clean-up method in which methanol was used once to extract melamine from the feed samples. No other clean-up operation was required. The operation time was reduced from 5 min to 2 min. The simple and fast clean-up operation is possibly related to the high selectivity and sensitivity of the AgNR array substrates.

**Fig 5:**
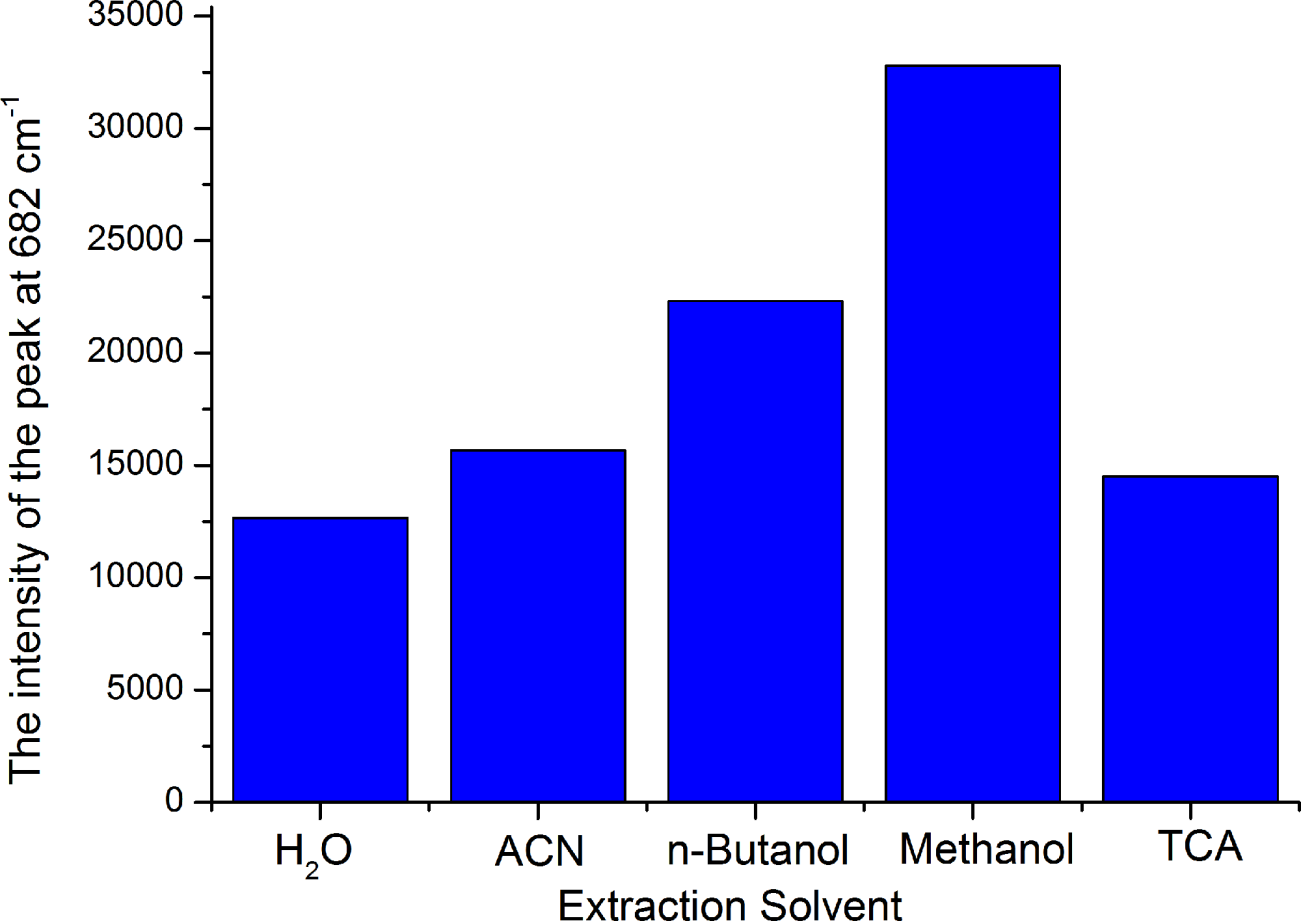
Intensity of the peak at △*ν* = 682 cm^−1^ using different extraction solvents.

Different reagents, such as H_2_O [29], 50% (v/v) acetonitrile in water [30], butyl alcohol [20], and trichloroacetic acid [33], have been used as melamine extraction solvents in previous studies. Along with these solvents, we also tested methanol as a melamine extraction reagent using the method outlined in the “Sample pretreatment” section, with 20 μg·g^−1^melamine added to the feed. Fig 5 shows that the intensity at △ν = 682 cm^−1^from the four previously tested extraction reagents are relatively weaker than methanol. Furthermore, methanol is able to solubilize and selectively extract melamine without matrix interference, as well as potentially remove interfering compounds, such as free amino acids, protein molecules, and pigments. Methanol was therefore chosen as the extraction solvent. As we known, the GC/MS method was the confirmatory methods, which need the solid-phase extraction cleanup and derivation operation. Compared with the GC/MS [11] method, the SERS analysis only need one step pre-treatment which save a lot of time.

### Impact of the incident laser energy

**Fig 6:**
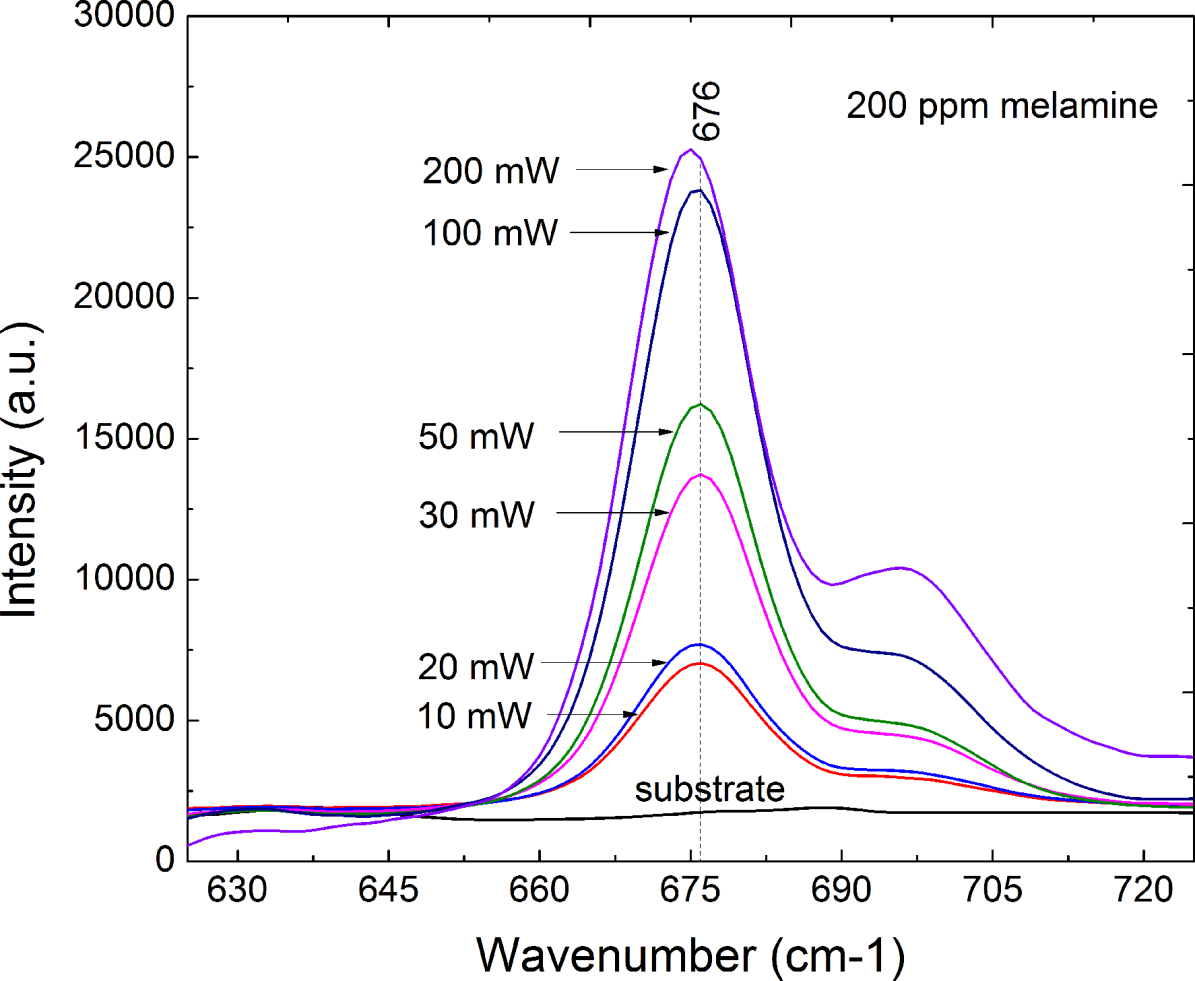
Optimization of incident laser power. Intensity of the characteristic peak at △*ν* = 676 cm^−1^as the incident laser power is increased from 10 mW to 200 mW.

Many methods are known to improve SERS signals, such as: (1) using a substrate with a larger enhancement to increase the average SERS signal of individual molecules, (2) increasing the laser power, and (3) using a substrate with a larger surface area to increase the maximum number of molecules producing the signal [34]. As mentioned above, we attempted to adjust the incident laser energy to increase the intensity of the characteristic melamine peak. Fig 6 shows that as the incident laser power is increased from 10 mW to 200 mW, the intensity of the characteristic peak at △*ν* = 676 cm^−1^ also increases. However, when the incident power is greater than 100 mW, the substrate is destroyed because of the excessive laser energy. Therefore, 100 mW is the optimal incident laser power.

### Stability of the AgNR array substrates

It is well known that AgNR array substrates easily suffer from oxidation [35]. Therefore, before each SERS measurement, we cleaned the substrate surfaces using HNO_3_ solvent (10^−7^ M) and methanol, which is of great importance to the entire operation. For the AgNR array substrate stability study, six batches of freshly fabricated AgNR array substrates were stored in a dryer at room temperature for 1, 2, 3, 4, 5, and 6 months. Each month, six repeat SERS measurements of the melamine standard solution (10 μg·mL^−1^) on the AgNR array substrates were performed. The same measurement protocol for SERS analysis was used as detailed above.

**Fig 7:**
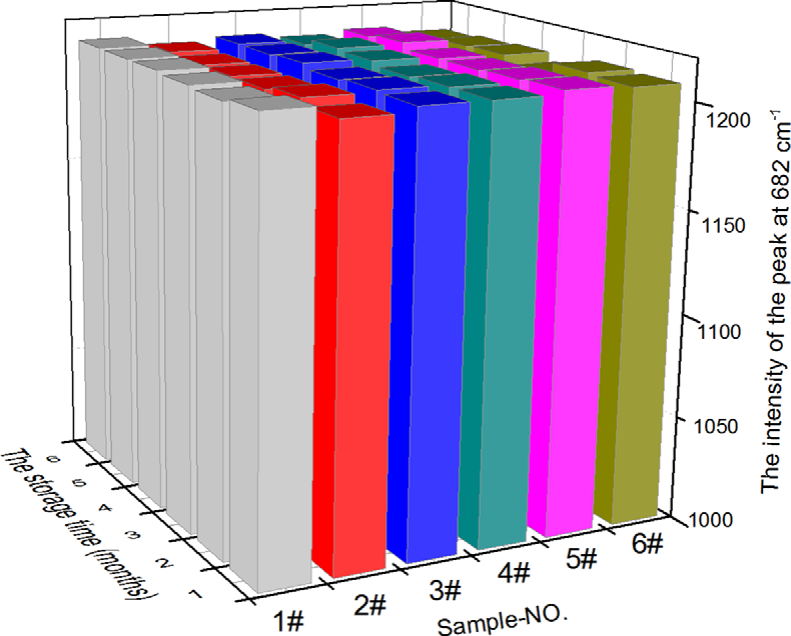
Stability of the AgNR array substrates (washed byHNO_3_ solvent and methanol). Comparison of different storage times versus intensity of the melamine Raman shift at △*ν* = 682 cm^−1^.

Fig 7 shows the relationship between the storage time and intensity of the melamine Raman shift at △*ν* = 682 cm^−1^. As the storage time increases from 1 to 6 months, the intensity of the characteristic melamine signal remains almost unchanged, indicating the good stability of the AgNR array substrates. In the previously reported method [20], the AuNPs colloids substrate was relatively stable under 4°C within 4 weeks. Compared with the AuNPs colloids substrate, the AgNR array substrates here showed more stable characteristics.

### Validation of the proposed method

#### Limit of detection (LOD) of melamine in real feed samples

The limit of detection (LOD) of melamine in the feed samples is the lowest concentration at which the peak intensity of the feed samples at △*ν* = 682 cm^−1^ is significantly different from the blank feed (control samples). The LOD was determined using the method from our previous study [18]. Briefly, at least nine spectra from two different blank feed substrates were measured. The peak intensities at △*ν* = 682 cm^−1^ of the blank feed were recorded. The average value plus three times the standard deviation of the blank feed was set as the limit for identifying a positive sample [35]. Therefore, when the sample spectrum intensity at △*ν* = 682 cm^−1^ is greater than the limit value, the sample is positive. Similarly, the limit of quantification (LOQ) was calculated from the average value plus ten times the standard deviation of the blank feed. By decreasing the melamine concentration stepwise, we measured the blank samples spiked with different concentrations and validated the sample clean-up method and the peak intensity response at △*ν* = 682 cm^−1^. By adjusting the concentration, the LOD (lowest concentration that yielded all positive detections) was ~0.9 μg·g^−1^ and the LOQ was 2 μg·g^−1^ which was more sensitive than the literature reported [36]. But compared with the LOD of my previously reported method (0.5 μg·g^−1^) [20], it was less sensitive. But the sample treatment was more simple and the LOD and LOQ values here could meet the maximum residual limit (2.5 μg·g^−1^) established by the European Union (EU) and China.

#### Linearity

The SERS spectra for different concentrations of feed samples spiked with known concentrations of melamine (2, 10, 50, 100, and 200 μg·g^−1^) (*n* = 6) were analyzed, and the SERS spectra are shown in Fig 3. A melamine fortified feed curve was obtained by establishing a plot correlating the melamine concentrations to the peak intensity at △*ν* = 682 cm^−1^. Linear regression (R^2^ = 0.926) is observed between the peak intensity at △*ν* = 682 cm^−1^ and spiked concentrations of melamine in the fortified feed (Fig 4) over the range 2–200 μg·g^−1^. The intensity of the characteristic peak at △*ν* = 682 cm^−1^ experiences a non-linear rise as the melamine concentration increases beyond 200 μg·g^−1^, which agrees with the results of previous studies [32,37]. When the concentration of the adsorbed melamine molecules increases to 200 μg·g^−1^, the surface coverage of the AgNRs reaches saturation and the peak intensity at △*ν* = 682 cm^−1^ does not increase linearly. However, when the adsorbed concentration is in the range 2–200 μg·g^−1^, the surface coverage of the melamine molecules is sufficient to form a monolayer if a uniform coverage is assumed and the peak intensity at △*ν* = 682 cm^−1^increases linearly. During analysis, if the signal response exceeded 200 μg·g^−1^, the sample was diluted appropriately and retested.

#### Recoveries and intraday and interday precisions

Melamine recovery experiments were performed. The blank feed samples spiked with 2, 6, 8, and 10 μg·g^−1^ melamine showed recoveries of 93.3, 92.5, 89.7, and 92.1%, respectively (Table 1). These results indicate the reliability of the method, which is acceptable based on EU recommendations [9].

**Table 1:** Recovery of Melamine from Spiked Feed Samples.

Concentration of melamine spiked in feed samples (μg·g^−1^)	Average intensity of the peak at 682 cm^−1^	Amount found^a^ (μg·g^−1^)	Recovery (%)(*n* = 5)	RSD^b^(%)
2	248.6	1.87 ± 0.03	93.3	2.02
6	728.4	5.55 ± 0.10	92.5	1.80
8	1003.2	7.18 ± 0.08	89.7	1.08
10	1202.2	9.21 ± 0.13	92.1	1.38

^a^Average values of five measurements for each concentration; Amound found value = Mean ± SD (Standard Deviation)

^b^Relative standard deviation of peak intensity (RSD (%) = (SD/mean) × 100).

Interday and intraday precision studies were performed for feed samples spiked with 2 μg·g^−1^melamine. Table 2 shows the relative standard deviation (RSD) values used to evaluate the repeatability of the method, which ranged from 0.14 to 0.16%. Thus, the results show good repeatability.

**Table 2:** Interday and Intraday Measurements to Assess the Repeatability of the Method.

Analysis^a^	Measurements	Spiked concentration (μg·g^−1^)	Amount found^b^ (μg·g^−1^)	Average intensity^a^ of the peak at 682 cm^−1^	Precision as RSD^b^(%)	RSD^c^(%)
**Intraday**	Day 1	5	4.90 ± 0.11	621.5	2.2	0.16
	Day 1	5	4.85 ± 0.09	618.4	1.8	
	Day 1	5	4.92 ± 0.11	628.3	2.3	
	Day 1	5	5.01 ± 0.14	631.4	2.8	
	Day 1	5	4.91 ± 0.11	622.3	2.3	
**Interday**	Day 1	5	4.95 ± 0.11	629.9	2.2	0.14
	Day 2	5	4.92 ± 0.13	628.5	2.6	
	Day 3	5	5.08 ± 0.16	631.5	3.1	
	Day 4	5	4.91 ± 0.11	619.3	2.3	
	Day 5	5	4.96 ± 0.12	629.7	2.4	

^a^Average of three repetitions for each measurement.

^b^Relative standard deviation (RSD (%) = (SD/mean) × 100) of individual measurements or days.

^c^RSD% of intraday and interday measurements.

#### Application of the proposed method

To investigate the proposed method, forty samples of the five types of listed feed (obtained from the Chinese National Feed Supervision from 2012–2014) were tested using the SERS method reported in this study. Table 3 shows the results of the AgNR array SERS method, which were validated and corroborated by GC/MS [9] and the colloidal Au SERS method reported previously [18]. Among three methods, we chose the result of GC/MS analysis as the standard. The GC/MS method was From the Table 3, we could find the results of the SERS method discussed here are more accurate than those obtained by the previous SERS method [18].

**Table 3:** Comparison of Results Obtained by GC/MS and SERS in 40 Feed Samples.

Sample Number	GC/MS (μg·g^−1^)	SERS		Quality^c^
		Quantity^a^ (μg·g^−1^)	Quantity^b^ (μg·g^−1^)	
**BCT20120175**	0.13	ND	ND	Negative
**BCT20120187**	ND^d^	ND	ND	Negative
**BCT20120190**	ND	ND	ND	Negative
**BCT20120191**	ND	ND	ND	Negative
**BCT20121167**	ND	ND	ND	Negative
**BCT20121365**	ND	ND	ND	Negative
**BCT20122189**	ND	ND	ND	Negative
**BCT20122190**	10.78	11.23	10.70	Positive
**BCT20122230**	ND	ND	ND	Negative
**BCT20122470**	ND	ND	ND	Negative
**BCT20130024**	ND	ND	ND	Negative
**BCT20130037**	ND	ND	ND	Negative
**BCT20130098**	44.38	46.33	45.09	Positive
**BCT20130120**	0.78	ND	ND	Negative
**BCT20130123**	ND	ND	ND	Negative
**BCT20130124**	ND	ND	ND	Negative
**BCT20130154**	ND	ND	ND	Negative
**BCT20130167**	ND	ND	ND	Negative
**BCT20130169**	ND	ND	ND	Negative
**BCT20130180**	ND	ND	ND	Negative
**BCT20130205**	ND	ND	ND	Negative
**BCT20130207**	ND	ND	ND	Negative
**BCT20130229**	ND	ND	ND	Negative
**BCT20130291**	ND	ND	ND	Negative
**BCT20130301**	19.61	20.98	20.01	Positive
**BCT20130322**	12.09	13.22	12.33	Positive
**BCT20130341**	ND	ND	ND	Negative
**BCT20130342**	ND	ND	ND	Negative
**BCT20130343**	ND	ND	ND	Negative
**BCT20130344**	ND	ND	ND	Negative
**BCT20130358**	68.90	70.01	69.41	Positive
**BCT20130366**	ND	ND	ND	Negative
**BCT20140008**	6.70	7.52	6.51	Positive
**BCT20140020**	ND	ND	ND	Negative
**BCT20140023**	ND	ND	ND	Negative
**BCT20140024**	ND	ND	ND	Negative
**BCT20140034**	ND	ND	ND	Negative
**BCT20140050**	ND	ND	ND	Negative
**BCT20140053**	ND	ND	ND	Negative
**BCT20140180**	ND	ND	ND	Negative

^a^Method of previous study [18].

^b^Method described in this study.

^c^A negative result means the content of melamine in the sample is below a concentration of 2.5 μg·g^−1^.

^d^ND (not detected) means the melamine content is below the LOD of the method (0.05 μg·g^−1^).

## Conclusions

In this study, we calculated the Raman spectra of melamine by DFT. The experimental Raman and SERS spectra of melamine correspond well with the DFT-calculated spectra. SERS detection using highly uniform AgNR array substrates was conducted for the detection of melamine from real feed samples. The method demonstrates high sensitivity (as low as 0.9 μg·g^−1^). Compared with the previously reported method, the modified clean-up method in this study reduced the detection time from 5 to 2 min and the active substrate was much more controllable and stable. This new protocol could be developed for rapid onsite screening of melamine contamination, for the purposes of quality control and market surveillance. Admittedly, in spite of the simplicity and rapidity of SERS, some mechanisms need to better evaluated, such as the discussion of potential interference in the feed, why the AgNR substrate could selectively enhance the Raman signal of melamine molecule. The research about the enhanced mechanism will be carried out in the future.

## Supporting Information

S1 Text The morphological characterization of AgNR. (DOCX)

S2 Text Raman and SERS peak assignments for melamine based on DFT calculations and experimental data. (DOCX)

S1 Fig The top-view (up) and cross-section (down) SEM images of Ag nanorod arrays with length L≈ 900 nm prepared at *θ* = 86°. (TIF)

S2 Fig The SERS detection flow diagram. (TIF)

S1 Table Band assignments for the DFT-Raman, experimental Raman, and SERS spectra of melamine. (DOCX)
