# Supplementary material for: Highly Sensitive Detection of Melamine Using a One-Step Sample Treatment Combined with a Portable Ag Nanostructure Array SERS Sensor
Source: PLoS One. 2016 Apr 27;11(4):e0154402. doi: 10.1371/journal.pone.0154402 (PMC4847794; doi:10.1371/journal.pone.0154402)
Supplement: S1 Text — (DOCX) [file pone.0154402.s001.docx]

**S1 Text.** **The morphological characterization of AgNR**

The morphologies of as-deposited samples were characterized by a field-emission scanning electron microscope (SEM) (JEOL, JSM 6510). Figure S1 show the top-view and cross-section SEM images of Ag nanorod array prepared at *θ*=86°. The vapor deposition angle θ is the angle between the vapor flux direction and the substrate surface normal. During the evaporation, the thickness of the metal deposited was monitored by a quartz crystal microbalance (QCM) positioned at normal incidence to the vapor source. The QCM reading length *l* is about 2310 ± 50 nm and the measured nanorod length L **≈** 900 nm for *θ*=86°. The Ag nanorod tilting angle *β* is defined as the angle between the Ag nanorod tilting direction and substrate surface normal. The nanorod tilting angle *β* can be directly measured from the cross-sectional SEM images, as shown in Figure S1 (*β* = 73°).
