# Supplementary material for: Highly Sensitive Detection of Melamine Using a One-Step Sample Treatment Combined with a Portable Ag Nanostructure Array SERS Sensor
Source: PLoS One. 2016 Apr 27;11(4):e0154402. doi: 10.1371/journal.pone.0154402 (PMC4847794; doi:10.1371/journal.pone.0154402)
Supplement: S2 Text — (DOCX) [file pone.0154402.s002.docx]

**S2 Text.** **Raman and SERS peak assignments for melamine based on DFT calculations and experimental data**

The major Raman peaks calculated by DFT (Supplementary S1 Table) agree well with the bulk Raman spectra acquired for melamine powder (Fig. 1). The most significant peaks observed in the DFT Raman spectra, viz. △ν=672 cm^−1^ and △ν = 1647 cm^−1^, are assigned to ring symmetrical stretching and H_14_–N_9_–H_15_ scissoring, respectively. However, several noticeable discrepancies between the DFT-calculated and experimental spectra are observed. For example, the calculated peaks at △ν=939, 1310, 1563, 1577, 1647, and 1684 cm^−1^ are missing in the corresponding experimental Raman spectrum. This is likely because DFT has an inherent tendency to overestimate the number of vibrational modes, possibly resulting from the tendency to neglect the an harmonicity present in real systems [1]. In addition, peaks with extremely low intensities could fall below the detection limit of the instrument at a given incident laser power and wavelength. Moreover, adjacent peaks estimated by DFT can emerge as a convoluted multipeak in experimental spectra, as observed with the peaks at △ν=1563 and 1577 cm^−1^, due in part to the low spectral resolution of the instrument. Furthermore, the peak intensities obtained from DFT appear to be different from the measured bulk Raman spectra, particularly in the △ν=522–587 cm^−1^ and △ν=1647–1691 cm^−1^ regions. In fact, similar observations have been reported in previous studies, which were attributed to preferencesfor certain vibrational modes during DFT calculations and the influence of molecular conformations in the crystalline structure [1].

**Reference:**

1. Wu XM, Gao SM, Wang JS, Wang HY, Huang YW, Zhao YP. The surface-enhanced Raman spectra of aflatoxins: spectral analysis, density functional theory calculation, detection and differentiation. Analyst. 2012; 137: 4226.
