# Supplementary figures and images for: Highly Sensitive Detection of Melamine Using a One-Step Sample Treatment Combined with a Portable Ag Nanostructure Array SERS Sensor
Source: PLoS One. 2016 Apr 27;11(4):e0154402. doi: 10.1371/journal.pone.0154402 (PMC4847794; doi:10.1371/journal.pone.0154402)

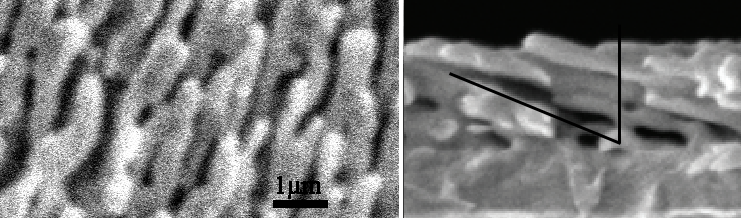

Supplement: S1 Fig — (TIF) [file pone.0154402.s003.tif]

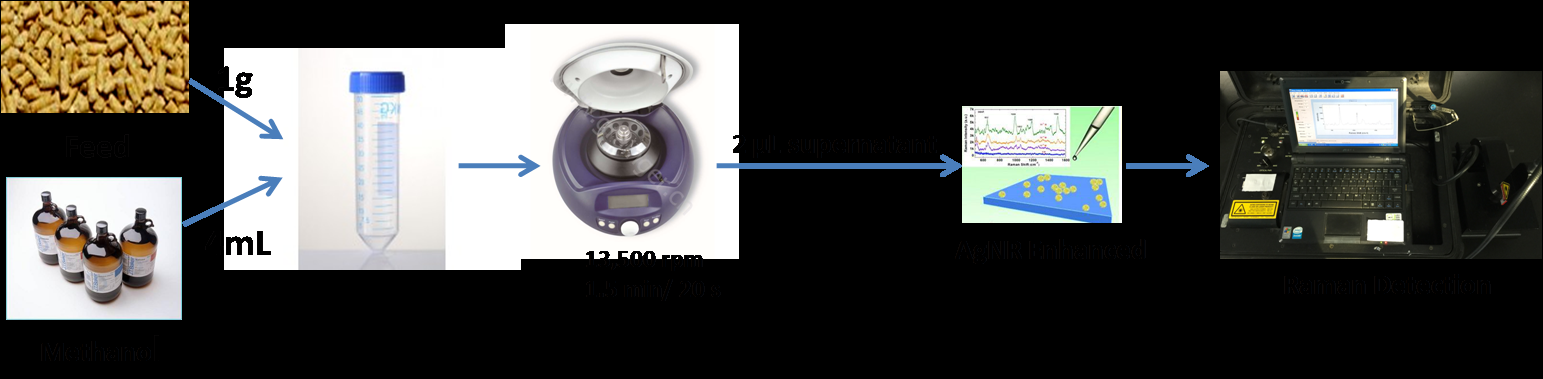

Supplement: S2 Fig — (TIF) [file pone.0154402.s004.tif]
