# Supplementary material for: Highly Sensitive Detection of Melamine Using a One-Step Sample Treatment Combined with a Portable Ag Nanostructure Array SERS Sensor
Source: PLoS One. 2016 Apr 27;11(4):e0154402. doi: 10.1371/journal.pone.0154402 (PMC4847794; doi:10.1371/journal.pone.0154402)
Supplement: S1 Table — (DOCX) [file pone.0154402.s005.docx]

**S1 Table. Band assignments for the DFT-Raman, experimental Raman, and SERS spectra of melamine**

| **DFT-**  **Raman (cm^-1^)** | **Experimental Raman (cm^-1^)** | **Experimental**  **SERS (cm^-1^)** | **Vibrational Mode** |
| --- | --- | --- | --- |
| 522 | 530 | - | N_8_–H_13_& N_7_–H_10_ wagging |
| 560 | 564 | 571 | H_14_–N_9_–H_15_ twisting |
| 581 | 587 | 587 | Ring stretching |
| - | - | 607 |  |
| 672 | 682 | 678 | Ring symmetrical stretching |
| 939 | - | - | Ring stretching |
| 971 | 992 | 993 | H_12_–N_8_–H_13_& H_11_–N_7_–H_10_rocking |
| 1175 | 1185 | 1177 | C_1_–N_9_& N_5_–C_1_–N_6_stretching |
| - | - | 1223 |  |
| 1310 | 1337 | - | Ringasymmetrical  stretching |
| 1475 | - | 1472 | C_1_–N_9_stretching |
| - | 1531 | - | C_1_–N_9_& C_2_–N_8_& C_3_–N_7_stretching |
| 1552 | - | 1557 | Ring asymmetrical  stretching |
| 1563 | - | - | C_1_–N_9_& C_2_–N_8_& C_3_–N_7_stretching |
| 1577 | - | - | C_2_–N_5_& C_3_–N_6_stretching |
| - | 1624 | 1621 | N_4_–C_3_–N_6_ Asymmetrical  stretching |
| 1647 | 1655 | - | H_14_–N_9_–H_15_scissoring |
| 1684 | 1691 | - | C_1_–N_9_& C_2_–N_8_& C_3_–N_7_stretching |
